# Living apart-together: Microhabitat differentiation of cryptic nematode species in a saltmarsh habitat

**DOI:** 10.1371/journal.pone.0204750

**Published:** 2018-09-27

**Authors:** Rodgee Mae Guden, Anna-Maria Vafeiadou, Nele De Meester, Sofie Derycke, Tom Moens

**Affiliations:** 1 Ghent University, Marine Biology Lab, Ghent, Belgium; 2 Mindanao State University- Iligan Institute of Technology, Iligan, Philippines; 3 Aristotle University of Thessaloniki, Biology Department, Thessaloniki, Greece; 4 Institute for Agricultural and Fisheries Research, Aquatic Environment and Quality, Oostende, Belgium; Hellenic Center for Marine Research, GREECE

## Abstract

Coexistence of highly similar species is at odds with ecological theory of competition; coexistence, then, requires stabilizing mechanisms such as differences in ecological niche. In the bacterivore nematode *Litoditis marina* species complex, which occurs associated with macro-algae, four cryptic lineages (Pm I-IV) co-occur in the field along the south-western coast and estuaries of The Netherlands. Here we investigate the temporal and/or spatial niche differentiation in their natural environment using a qPCR-based detection and relative quantification method. We collected different algal species (i.e. two *Fucus* species and *Ulva* sp.) and separated algal structures (i.e. receptacula, thalli, non-fertile tips and bladders) at different sampling months and times (i.e. twice per sampling month), to examine differences in microhabitat use between coexisting *L*. *marina* species. Results demonstrate that the cryptic species composition varied among different algal species and algal structures, which was also subject to temporal shifts. Pm I dominated on *Fucus* spp., Pm II showed dominance on *Ulva* sp., while Pm III overall had the lowest frequencies. Microhabitat partitioning was most pronounced between the two cryptic species which had similar microbiomes (Pm I and Pm II), and less so between the two species which had significantly different microbiomes (Pm I and Pm III), suggesting that species which share the same microhabitats may avoid competition through resource partitioning. The interplay of microhabitat differentiation and temporal dynamics among the cryptic species of *L*. *marina* implies that there is a complex interaction between biotic components and abiotic factors which contributes to their coexistence in the field.

## Introduction

Most estimates of biodiversity rely on inventories of morphologically identifiable species. In the past two decades, the prominence of cryptic species, i.e. morphologically (nearly) identical but genetically distinct species, in many taxa has challenged existing estimates of biodiversity [1, 2]. Cryptic diversity is common across phyla and biogeographic regions [3] and may be particularly prominent in marine habitats, where many species rely on chemical signals for mating and for ecological interactions [4–6]. Interspecific differences in these non-visual traits hardly leave a morphological imprint [1]. Multiple cryptic species can also coexist at local scales [7–9], which is at odds with our traditional view of ecological competition theory since strong interspecific competition is expected between species occupying the same ecological niche [10–12]. With neutral dynamics, ecologically similar species are able to coexist for extended periods of time as their relative abundances change through a completely stochastic drift process [13,14]. Nevertheless, species with high phenotypic similarity can also strongly differ in ecologically significant means, for instance in behaviour and physiology or in life history traits, which may lead to utilization of different niches [15,16], suggesting that traditional niche partitioning mechanisms may be enough in at least some communities to promote stable coexistence [17].

Cryptic diversity has also repeatedly been detected in marine nematodes [18–21]. Nematodes are one of the most abundant and diverse faunal groups in marine environments [22,23]. They may play significant roles in the functioning of estuarine and marine ecosystems, since they are involved in benthic mineralization and decomposition processes [24,25]. They are also useful ecological indicators since they can be assigned to different trophic or functional groups with varying sensitivities to pollutants and disturbance [26–28]. The presence of cryptic diversity implies that some species which have long been considered ecological generalists, and/or as having a broad geographical distribution, may instead be groups of more ecologically specialized and/or geographically more restricted taxa [29]. Assessing the ecology of nematodes may therefore lead to inappropriate interpretations if cryptic species with potentially different responses to environmental conditions and relationships with other organisms, are not recognized [30]. This may have important repercussions for the use of nematodes as bioindicators, since different cryptic species may have distinct responses to pollutants [31; see also 32,33 for examples from other invertebrate phyla] and possibly unique functional roles [34]. Therefore, a comprehensive understanding of cryptic diversity is indispensable.

*Litoditis marina* [35], formerly known as *Rhabditis marina* [36] or *Rhabditis* (*Pellioditis*) *marina* [37], is the best studied marine nematode species complex in the pursuit to understand cryptic diversity. It is a bacterivore nematode associated with living and decomposing macroalgae in the littoral zone of coasts and estuaries [38]. At least 10 cryptic lineages have already been discovered in the *L*. *marina* complex [19]. Furthermore, the sympatric distribution of multiple cryptic species is common; for instance, at least three of the four most abundant cryptic species of *L*. *marina* along the south-western coast of The Netherlands often co-occur in the field [18,39]. The mechanisms promoting the co-occurrence of cryptic species of *L*. *marina* at a local scale have been extensively investigated over the past decade. The timing of abundance peaks of the different cryptic species fluctuates over the course of a year [39]. *L*. *marina* are typical colonizers of decomposing macroalgae and manifest strong colonization-extinction dynamics [40]. In laboratory experiments, differential dispersal strategies among these cryptic species were observed [41]. Abiotic factors such as temperature and salinity differentially influence their dispersal abilities [41], life histories, reproductive strategies [42], and interspecific interactions [43,44], which may indicate different forms of niche differentiation as a mechanism for the coexistence of these cryptic species. Differential food preferences and feeding behaviour, as assessed by characterizing bacterial communities associated with field-collected specimens using next generation sequencing of the microbial 16S rRNA gene and by performing laboratory trials, are other potential factors which can promote niche differentiation in the *L*. *marina* species complex [45]. However, differences in microhabitat use of the cryptic species of *L*. *marina* in their natural environment have not been explored yet. *L*. *marina* consistently occurs in the intertidal area of the Paulina tidal flat and salt marsh in the Schelde Estuary, The Netherlands. This area is a very heterogeneous environment which shows temporal (daily to seasonal) and spatial abiotic fluctuations [46]. The Paulina area also encompasses algal holdfasts on stones and on a breaker, as well as deposits of dead algae, particularly higher up in the marsh. The most abundant intertidal seaweeds here belong to the genera *Fucus* and *Ulva*, which offer suitable habitat to *L*. *marina* [38]. These macroalgae are often covered with microbial biofilms and have different structural features; hence, they represent a heterogeneous environment in their own right.

**Fig 1:**
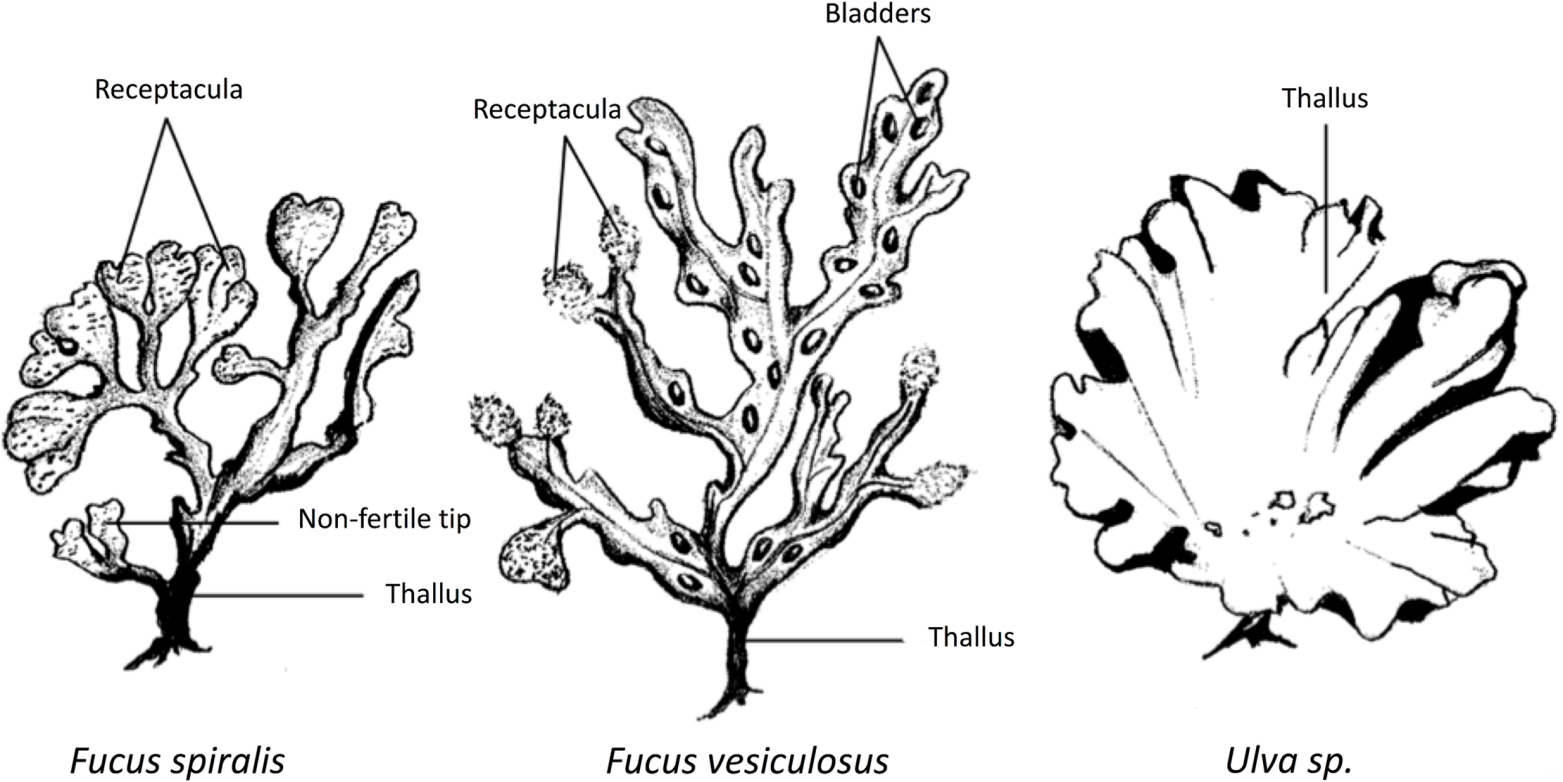
Structures of *Fucus spiralis*, *Fucus vesiculosus* and *Ulva* sp. collected in the Paulina intertidal area.

In this study, different macroalgae species, i.e. *Fucus spiralis*, *Fucus vesiculosus*, and *Ulva* sp., were collected from the Paulina area to investigate temporal and spatial variability in microhabitats for *L*. *marina* cryptic species. Different structures of *F*. *spiralis* and of *F*. *vesiculosus* (Fig 1) were examined. Samplings were conducted in November and April to determine temporal variation. We hypothesized that the cryptic species of *L*. *marina* would occupy at least partly different microhabitats. Specifically, we tested whether cryptic species composition of *L*. *marina* differed a) among species of algae; b) among different structures of a specific algal species, here *F*. *spiralis* and *F*. *vesiculosus*; and c) between months. Furthermore, we assessed whether the occurrence of each cryptic species on particular algal species and structures was consistent over sampling times within a particular month; for this purpose, we sampled twice with a 2-week interval within each month. Differential microhabitat may help to explain the co-occurrence of *L*. *marina* cryptic species complex and support the idea that niche partitioning is a significant process promoting coexistence.

## Materials and methods

### Sampling location and sampled macroalgae

Samplings were conducted in November 2015 and April 2016 along the edges of the Paulina salt marsh area (51° 20' 56" N, 3° 43' 29" E), which is in the polyhaline reach of the Schelde Estuary, The Netherlands, to determine the relative abundances of four cryptic species of *L*. *marina* (Pm I, Pm II, Pm III and Pm IV). Within both months, two samplings were performed with a 2-week interval to determine if patterns were consistent over time. We did not quantify absolute abundances of *L*. *marina*, which would have been very difficult to compare between different algae and algal structures because we could not properly standardize the amount of sample across all algal specimens and structures. Furthermore, other nematode species were also present in the samples, rendering it highly time-consuming to count and identify *L*. *marina*.

*Fucus vesiculosus* and *Fucus spiralis*, which are the most common macroalgae in the Paulina area and are among the most common habitats of *L*. *marina* based on earlier observations at this location, were randomly collected, always with at least six replicates per species. Both ‘clean’ and sediment-covered *Fucus* spp. were collected. ‘Clean’ algae were not prominently covered with biofilms, whereas sediment-covered algae had prominent biofilms with sediment embedded (Table 1). Each replicate was one whole living plant attached to rocks and/or dikes. *Fucus spiralis* lacks bladders with swollen receptacles and a flattened blade while *F*. *vesiculosus* is mainly characterized by the presence of round-shaped bladders which are usually paired and occur on either side of a conspicuous midrib [47] (Fig 1). Thalli of *Ulva* sp., which were sparsely deposited on the sediment, were also collected with three to six replicates.

**Table 1:** Summary of the collected samples (number of replicates for every sampling time x algal species x sample type) in the Paulina area in November and April. ‘Clean’ algae were not prominently covered with biofilms, whereas algae ‘with sediment’ had prominent biofilms with sediment embedded. In November, clean *Fucus vesiculosus* were very rare and therefore were not sampled.

Algal species	Type	November		April	
		1^st^ sampling	2^nd^ sampling	1^st^ sampling	2^nd^ sampling
*Fucus spiralis*	with sediment	4 replicates	6 replicates	6 replicates	6 replicates
	clean	6 replicates	6 replicates	6 replicates	6 replicates
*Fucus vesiculosus*	with sediment	6 replicates	6 replicates	6 replicates	6 replicates
	clean	none	none	6 replicates	6 replicates
*Ulva* sp.		6 replicates	3 replicates	4 replicates	4 replicates

No specific permissions were required for the described field investigation: the sampling location is not privately-owned or protected in any way and the field study did not involve endangered or protected species.

### Processing of macroalgae

The different structures of *F*. *spiralis* (receptacula, thalli and non-fertile tips) and *F*. *vesiculosus* (receptacula, thalli and bladders) were immediately separated in the lab by cutting them with scissors, and all the algal structures were frozen at -20°C until they were processed. Processing of macroalgae involved washing the separated parts of *Fucus* spp. and thalli of *Ulva* sp. in 1 L of distilled water which was then poured onto a stack of two sieves with respective mesh sizes of 120 and 32 μm to separate adult from juvenile nematodes. Separating the fractions of both stages was conducted to achieve higher precision for quantification since adults and juveniles differ in cell numbers [48]. The fractions retained on each sieve were transferred to separate 50 ml falcon tubes, and then diluted up to a volume of 50 ml. The nematodes were allowed to sink for 15 min on ice. Afterwards, the samples were centrifuged for 10 min at room temperature and maximum speed, with 0 breaking level to gently stop the centrifugation. The supernatant was removed, while the pellets were gradually transferred to a sterile 2 ml tube by centrifugation for 3 min at 10,000 rpm.

### DNA extraction

The DNA of the collected nematodes was extracted using hexadecyltrimethylammonium bromide (CTAB) according to the protocol modified for *Litoditis marina* [48]. 500 μl of CTAB buffer was added to each tube with the final nematode pellet (2% w⁄ v CTAB, 1.4 M NaCl, 0.2% (v⁄v) 2-mercaptoethanol, 20 mM EDTA, 100 mM TRIS ⁄ HCl pH 8.75), then frozen at -80°C. Subsequently, enzymatic and mechanical lysis were performed by adding 12 μl of proteinase K (10 mg μl^-1^) and 0.1 g of glass beads to the tubes, followed by bead beating for 30 s at 3000 cycles min^-1^ and incubation at 60°C for 1 h. Then, 500 μl of the upper layer of the solution was transferred to a new sterile 1.5 ml tube; 250 μl of 7.5 M ammonium acetate was added to dissolve the DNA. This was followed by DNA precipitation in 720 μl of cold isopropanol. The last step involved washing the DNA pellet with 1 ml washing buffer (76% ethanol and 10 mM ammonium acetate), followed by addition of 20 μl sterile water.

### Real-time quantitative PCR (qPCR)

Relative quantification of the four cryptic *L*. *marina* species was performed using qPCR of the ribosomal ITS region [48]. The qPCR assay was conducted using the LightCycler 480 System and the LightCycler 480 SYBR Green I master kit (Roche diagnostics). The qPCR mixtures were prepared for 10 μl volumes using a 384-well plate, which contained 3 μl of primer mix (final concentration of 1 μM for Pm I and Pm III, 500 nM for Pm II and 200 nM for Pm IV), 5 μl of master mix, 1.5 μl of water, and 0.5 μl of the DNA samples. Prior to this, the DNA concentration of the samples was measured with the Nanodrop 2000 (Isogen Life Science); concentrations above 10 ng μl^-1^ were diluted with distilled H_2_O to avoid inhibition effects when a high amount of DNA template is present. Each run of the qPCR involved a positive (with 0.5 μl of DNA template) and a negative (no DNA template) control, and all samples were run with two technical replicates. The thermal cycling protocol was composed of four steps involving an initial denaturation for 10 min at 95°C, 40 cycles of denaturation for 10 s at 95°C, annealing for 20 s at 60°C, then extension for 20 s at 72°C. In order to verify the amplification of the specific products and absence of primer dimers, C_t_ values within or above the cycle threshold (35) were discarded and a melting curve analysis was conducted using a temperature range of 65 to 97°C and an increase of 0.6°C s^-1^. Differences in C_t_ values were calculated using the adjusted ΔΔCT method [48,49] to determine the relative abundances of each cryptic species. A reference sample was used for the relative quantification [34]. This reference sample was comprised of 40 replicates pooled into one tube, each containing 25 specimens each of Pm I, Pm II, Pm III and Pm IV, making up a total of 100 nematodes in each tube. The C_t_ values were averaged across these replicates to obtain one C_t_ value for each cryptic species.

### Data analyses

The relative abundances of Pm I, Pm II, and Pm III (no Pm IV was found) were used for the analysis of cryptic species composition on different microhabitats with separate tests for adults and juveniles. All statistical tests were conducted using R [50]. Permutational Multivariate Analysis of Variance (PERMANOVA) was performed using the Adonis function (Vegan package) [51] on the basis of Euclidean distance with 999 permutations since the data were not normally distributed even after transformation. To determine whether the variation among replicates also influenced the differences in cryptic species composition, the betadisper command was used to test for the homogeneity of group dispersions (PERMDISP). Significant factors and interactions were analysed using posterior pairwise comparisons with a Bonferroni correction.

#### Effects of algal species, month and sampling time in cryptic species composition

A three-way PERMANOVA test with factors: algal species (3 levels), month (2 levels) and sampling time (4 levels) nested in month was used to determine the effects of each of these factors and their interactions in the cryptic species composition on different algal species. All three algal species, *F*. *spiralis*, *F*. *vesiculosus* and *Ulva* sp., were included in this analysis. Only the sediment-covered *Fucus* samples were used in the test because most of the clean samples did not carry any *Litoditis*. Given that no differentiation of structures was possible for *Ulva* sp., the comparison among algal species used the average of the relative abundances of nematodes on all algal structures of a single alga for each *Fucus* species.

#### Effects of algal structure, month and sampling time in cryptic species composition

To test for significant differences in cryptic species composition between the structures of *Fucus* species, analyses were performed separately for *F*. *spiralis* and *F*. *vesiculosus* using a three-way PERMANOVA test with factors: algal structures (3 levels), month (2 levels) and sampling time (4 levels) nested in month. The interactions of algal structures with month and with sampling time were also analysed to determine whether the observed differences were consistent across different months and different sampling times. Considering that the algal structures of each individual alga are linked, ID (specimen) was included in the model as a random variable.

## Results

Our results indicated that the cryptic species composition of the *L*. *marina* complex varied among different algal species and algal structures, patterns which were also subject to temporal shifts. We used the relative abundances of Pm I, Pm II, and Pm III to assess the cryptic species composition on different microhabitats. No Pm IV was found throughout our investigation. A summary of the collected samples indicating the number of replicates for every sampling time and algal species is presented in Table 1.

### Cryptic species composition varied among algal species and between months

**Fig 2:**
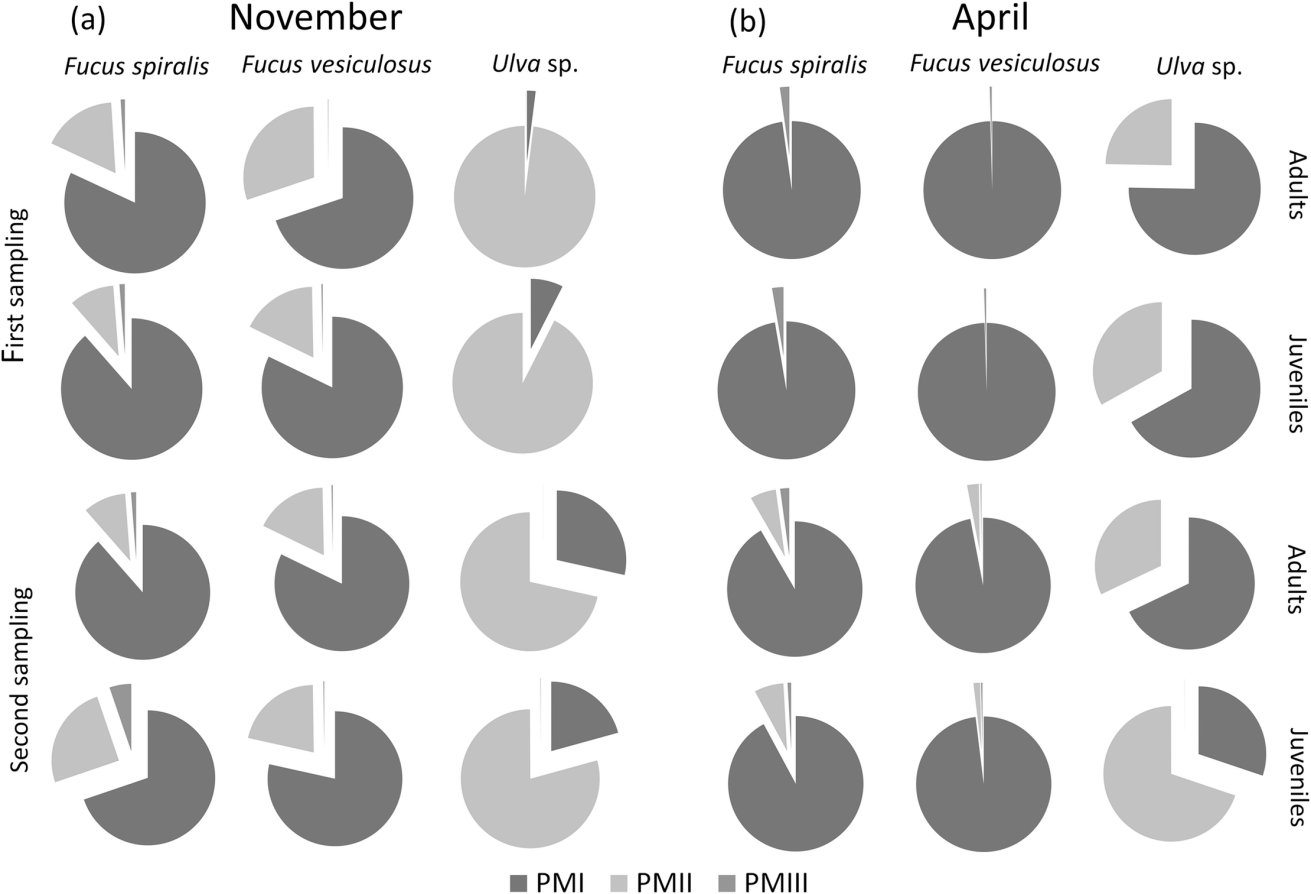
Average proportional abundances of Pm I, Pm II and Pm III adults and juveniles at two sampling times in (a) November and (b) April on *Fucus spiralis* (n = 6, except for the first sampling in November, where n = 4), *Fucus vesiculosus* (n = 6) and *Ulva* sp. (n ≥3). Proportions of *Fucus* spp. are an average of the relative abundances of *L*. *marina* on all algal structures.

The cryptic species composition of *L*. *marina* adults and juveniles was significantly different among algal species and between months. Pm I showed dominance on *F*. *spiralis* and *F*. *vesiculosus*; Pm II dominated on *Ulva* sp., while Pm III overall had the lowest frequencies and reached its highest relative abundances on *F*. *spiralis* (Fig 2). Algal species and month had significant effects on the species composition of *L*. *marina* for both stages (all *P* = 0.001), while the interaction of these two factors was only significant in adults (*P* = 0.002). Sampling time did not have a significant effect for both stages (Table 2, Fig 2).

**Table 2:** PERMANOVA results from the analysis of the proportions of Pm I, Pm II and Pm III adults and juveniles as a function of algal species, month and sampling time (nested in month). Significant differences (*P*<0.05) are highlighted in bold.

Source	Adults		Juveniles	
	F	*P* (perm)	F	*P* (perm)
Algal species	26.87	**0.001**	36.65	**0.001**
Month	20.2	**0.001**	27.99	**0.001**
Sampling time (month)	3.67	0.06	1.50	0.23
Month*sampling time (month)	1.11	0.29	0.05	0.94
Algal species*month	8.21	**0.002**	1.62	0.22
Algal species*sampling time (month)	0.34	0.75	0.90	0.42
Algal species*month*sampling time (month)	1.13	0.30	0.45	0.54

Pairwise tests on the significant effect of algal species for juveniles showed significant differences between *F*. *spiralis* and *Ulva* sp., and between *F*. *vesiculosus* and *Ulva* sp. (both *P* = 0.0003, Table 3). For adults, the significant interaction of algal species with month revealed that temporal differences in the cryptic species composition were not consistent across algal species and vice versa. Adult nematode composition was significantly different between *F*. *spiralis* and *Ulva* sp. (*P* = 0.0015), and between *F*. *vesiculosus* and *Ulva* sp. in November (*P* = 0.0015) but not in April (Table 3). The cryptic species composition was significantly different between November and April for *F*. *vesiculosus* (*P* = 0.006) and for *Ulva* sp. (*P* = 0.049) (Table 3). Pm I was consistently dominant on *F*. *spiralis* and *F*. *vesiculosus*. Pm II was dominant on *Ulva* sp. in November but had low relative abundances in April. Pm III had lower proportions compared to Pm I and Pm II and had relatively higher proportions on *F*. *spiralis* (Fig 2), while Pm IV remained absent throughout our study.

**Table 3:** Pairwise test results on the significant effect of algal species in the cryptic species composition for juveniles and on the significant interaction of algal species and month for adults. Significant differences (*P*<0.05) are highlighted in bold.

**Juveniles**	F	*P* value	*P* adjusted			
*F*. *spiralis*, *F*. *vesiculosus*	0.097	0.90	1.00			
*F*. *spiralis*, *Ulva* sp.	32.63	0.0001	**0.0003**			
*F*. *vesiculosus*, *Ulva* sp.	33.97	0.0001	**0.0003**			
**Adults**						
**Between algal species**	**November**			**April**		
	F	*P*	*P*	F	*P*	*P*
		value	adjusted		value	adjusted
*F*. *spiralis*, *F*. *vesiculosus*	3.99	0.056	0.85	2.41	0.13	1.00
*F*. *spiralis*, *Ulva* sp.	101.41	0.0002	**0.0015**	1.27	0.29	1.00
*F*. *vesiculosus*, *Ulva* sp.	85.72	0.0001	**0.0015**	2.72	0.11	1.00
**Between months**	F	*P*	*P*			
		value	adjusted				
*F*. *spiralis*	2.60	0.096	1.00				
*F*. *vesiculosus*	12.31	0.0004	**0.006**			
*Ulva* sp.	11.82	0.0033	**0.049**			

PERMDISP showed lack of homogeneity of variances for the significant interaction of algal species with month for adults (F = 15.34, *P* = 0.001); hence, careful interpretation is needed. This indicated that the cryptic species composition was not only influenced by algal species and month, but also by the variation among replicates.

### Cryptic species composition varied among algal structures and between months

**Fig 3:**
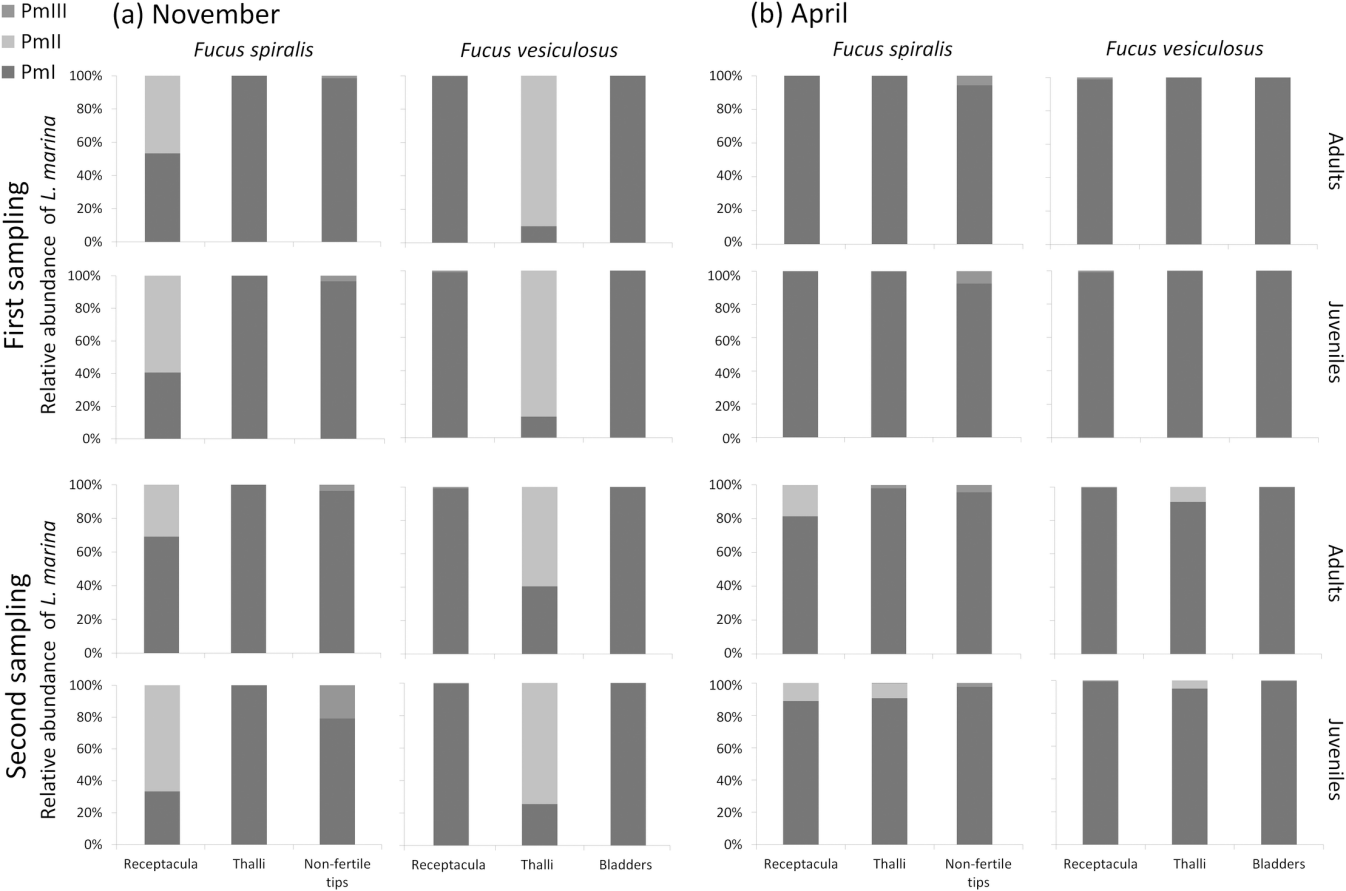
Average proportional abundances of Pm I, Pm II and Pm III adults and juveniles on different structures of *Fucus spiralis* (in order: receptacula, thalli and non-fertile tips; n = 6, except for the first sampling in November, where n = 4) and *Fucus vesiculosus* (in order: receptacula, thalli and bladders; n = 6) for the two sampling moments (n = 6) in (a) November and (b) April.

The cryptic species composition of *L*. *marina* varied among different structures of *F*. *spiralis* (receptacula, thalli and non-fertile tips) and *F*. *vesiculosus* (receptacula, thalli and bladders) for both stages of nematodes, and between months (Fig 3). On *F*. *spiralis*, Pm I was present on all algal structures, but was proportionally the most abundant on the thalli. In contrast, Pm II was only present on the receptacula and Pm III was found in higher proportions on the non-fertile tips. Only the algal structure had a significant effect in the cryptic species composition of adults (*P* = 0.015) on *F*. *spiralis*, while algal structure, month and the interaction of these two factors had significant effects for juveniles (all *P*<0.005, Table 4, Fig 3). On *F*. *vesiculosus*, Pm I largely dominated the receptacula and bladders, while Pm II had high proportions on the thalli, and Pm III had a higher relative abundance on the receptacula compared to other structures of *F*. *vesiculosus*. Algal structure, month and their interaction had significant effects in the cryptic species composition of both stages on *F*. *vesiculosus* (all *P* = 0.001, Table 4, Fig 3). Sampling time also had a significant effect for juveniles (*P* = 0.001) on *F*. *spiralis* and for both stages on *F*. *vesiculosus* (both *P* = 0.001), but a closer look through a pairwise test showed significant differences for sampling time between different months but not within the same month (S1 Table). Hence, the results were consistent over sampling times within a particular month.

**Table 4:** PERMANOVA results from the analysis of the cryptic species composition of adults and juveniles on *Fucus spiralis* and *Fucus vesiculosus* as a function of algal structures and month, with sampling time nested in month. Significant differences (*P*<0.05) are highlighted in bold.

***Fucus spiralis***	**Adults**		**Juveniles**	
**Source**	F	*P* (perm)	F	*P* (perm)
Algal structure	3.61	**0.015**	13.56	**0.001**
Month	2.71	0.078	8.64	**0.001**
Sampling time (month)	0.21	0.078	1.74	**0.001**
Algal structure*month	1.78	0.13	5.61	**0.002**
Algal structure*sampling time (month)	0.26	0.78	1.14	0.55
***Fucus vesiculosus***				
**Source**				
Algal structure	33.28	**0.001**	30.78	**0.001**
Month	9.87	**0.001**	22.74	**0.001**
Sampling time (month)	0.99	**0.001**	2.80	**0.001**
Algal structure*month	14.93	**0.001**	17.82	**0.001**
Algal structure*sampling time (month)	1.30	0.38	1.08	0.43

Pairwise tests on the significant effect of algal structure on *F*. *spiralis* for adults showed no significant p adjusted values (all p>0.05), implying that the differences in the cryptic species composition for adults on different structures of *F*. *spiralis* may not be strong enough although significant overall p-values were obtained (Table 5). For juveniles on *F*. *spiralis*, pairwise tests on the significant interaction of algal structure and month revealed variation between algal structures in November but not in April (Table 5). In particular, cryptic species composition was significantly different between the receptacula and thalli (*P* = 0.0045) and between the thalli and non-fertile tips (*P* = 0.0075). On *F*. *vesiculosus*, significant differences were found between the cryptic species composition on the receptacula and the thalli for adults and juveniles (both *P* = 0.0015) and between the thalli and the bladders (*P* = 0.0015) for adults in November but not in April (Table 5).

**Table 5:** Pairwise test results on the significant effect of algal structure in the cryptic species composition for adults on *Fucus spirali*s and on the significant interaction of algal structure and month for juveniles on *Fucus spirali*s and for both stages on *Fucus vesiculosus*. Significant differences (*P*<0.05) are highlighted in bold.

***Fucus spiralis***	F	*P* value	*P* adjusted			
**Adults**		* *	* *			
Receptacula vs thalli	3.73	0.049	0.14			
Receptacula vs non-fertile tips	4.80	0.024	0.072			
Thalli vs non-fertile tips	0.175	0.98	1.00			
	**November**			**April**		
***Fucus spiralis***	F	*P* value	*P* adjusted	F	*P* value	*P* adjusted
**Juveniles**						
Receptacula vs thalli	24.56	0.0003	**0.0045**	0.98	1.00	1.00
Receptacula vs non-fertile tips	10.49	0.0036	0.054	0.44	1.00	1.00
Thalli vs non-fertile tips	5.57	0.0005	**0.0075**	2.66	0.11	1.00
***Fucus vesiculosus***							
**Adults**						
Receptacula vs thalli	43.35	0.0001	**0.0015**	0.68	0.53	1.00
Receptacula vs bladders	4.16	0.008	0.12	0.01	0.64	1.00
Thalli vs bladders	43.88	0.0001	**0.0015**	0.75	0.46	1.00
**Juveniles**						
Receptacula vs thalli	60.79	0.0001	**0.0015**	0.05	0.91	1.00
Receptacula vs bladders	4.57	0.10	1.00	1.13	0.24	1.00
Thalli vs bladders	27.61	0.0001	**0.0015**	1.47	0.33	1.00

PERMDISP further revealed that the significant interaction of algal structures and species on *F*. *spiralis* for juveniles (F = 10.44, *P* = 0.001) and on *F*. *vesiculosus* for both adults and juveniles (F = 20.03 and 16.44 respectively, both *P* = 0.001) was also influenced by the variation among replicates (no homogeneity of variances).

## Discussion

Owing to the difficulties of distinguishing cryptic species, the use of molecular genetic methods such as real-time quantitative PCR offers a rapid, specific and sensitive approach for detecting and quantifying the cryptic species of *Litoditis marina* [48]. This requires the use of species-specific primers. The ITS region showed an appropriate level of variation between species while intraspecific differences remain low [19,52]. Moreover, the high variability of the ITS region between nematode species makes it unlikely for more distantly related species to be amplified with the primers [48]. Nevertheless, critical analysis is required to avoid overestimation of the target species, and thus be able to use this technique for field studies. Real-time quantitative PCR may even be extended to encompass nearly assemblage-wide analyses as it allows quantification of multiple specific species within communities [53,54].

In the Paulina intertidal area in the Schelde estuary, the sympatric distribution of two or more cryptic species within the *Litoditis marina* complex is rule rather than exception [39]. Here, we demonstrate that the cryptic species occur in partly different microhabitats (algal species and structures), which may play a role in this co-occurrence, and that the cryptic species composition in these microhabitats varies temporally. It is important, in this respect, to note that we report relative abundances throughout our study. We did not quantify absolute abundances of *L*. *marina* because obtaining properly standardized amounts of different algae and algal structures would have been extremely tedious. Furthermore, other nematode species were also present in the samples, and we chose not to enter the highly time-consuming task of identifying and counting all nematodes. As a consequence, we cannot draw firm conclusions on which microhabitats are ‘favoured’ or ‘preferred’ by particular cryptic species, and we may even have missed more ‘optimal’ habitats. However, our results are conclusive with respect to the differences in species composition on the algal species and structures documented here.

### Cryptic species composition varies among different algal species

The cryptic species composition of *L*. *marina* adults and juveniles varied among different algal species (Fig 2). Pm I dominated on *Fucus spiralis* and *F*. *vesiculosus*; Pm II showed dominance on *Ulva* sp., while Pm III overall had the lowest frequencies: it reached its highest relative abundances on *F*. *spiralis*. Pm IV was not detected in the Paulina area. The consistency of our results over different sampling times within each month indicates that the observed patterns were not just stochastic in nature. Moreover, they corroborate results of a seasonal study focusing on *Fucus* sp. (mostly *F*. *vesiculosus*) in the Paulina area, where Pm I was also by far the most abundant cryptic species, followed by Pm II; Pm III was rarer than both other species, and Pm IV was completely absent [39]. In another sampling area (Lake Grevelingen) in the same region, where only *Ulva* sp. algae were collected, Pm I was not found while Pm II was dominant with presence of Pm IV [39]. This supports our findings that different cryptic species of *L*. *marina* may prefer different algal species as a substratum, Pm I being more common on *Fucus* spp. and Pm II on *Ulva* sp. Since Pm IV often co-occur with Pm II on *Ulva* sp. in Lake Grevelingen, its absence from the Paulina area may reflect differences in environmental conditions, Lake Grevelingen having a fairly constant salinity and no tidal currents [18]. Habitat partitioning of cryptic marine species by environmental variables, such as salinity and wave action, has also been demonstrated in studies dealing with snails [55] and fishes [56].

Our results thus clearly demonstrate that the cryptic species composition of *L*. *marina* is influenced by algal species. *L*. *marina* mainly feeds on bacteria associated with macroalgae [57]. Indeed, we found lower frequencies of *L*. *marina* on clean than on biofilm-covered *Fucus* spp. (data not shown). In many cases, clean macroalgae did not carry any *Litoditis*. This indicates that the microbial biofilms on the surfaces of *Fucus* spp. offer a rich food source for *L*. *marina*. The biofilm-covered samples also had sediments embedded, which are likely sources of microbial recruitment on the surfaces of macroalgae [58]. The bacterial communities covering macroalgae are often host-specific [59–62]. Although microbial communities can vary among individuals of the same algal species [63], they are highly dissimilar between species (less so between closely related species) and even more so between phyla [60–61]. This may explain the observed stronger difference in the cryptic species composition between *Fucus* spp. and *Ulva* sp. than between *F*. *spiralis* and *F*. *vesiculosus*. The occurrence of Pm I on the two closely related species *F*. *spiralis* and *F*. *vesiculosus*, and that of Pm II on *Ulva* sp., may thus be linked to the difference in bacterial communities on the surfaces of these macroalgae. In fact, prominent presence of bacteria which are well represented on the surface of *F*. *vesiculosus* [61] was found in the microbiomes of *L*. *marina* [45]. Furthermore, the microbiomes of Pm I and Pm II obtained from the same *Fucus* algae were found to differ significantly, whereas the microbiomes of Pm I and Pm III did not significantly differ [45]. Our study demonstrated that microhabitat partitioning on different algal substrata was strongest between Pm I and Pm II, and least pronounced between Pm I and Pm III, suggesting a trade-off between microhabitat differences and resource partitioning. Pm I and Pm III, which tended to occupy the same algal species in our study, may avoid competition through resource partitioning, with Pm III being a more selective feeder than Pm I [45]. Other aspects of the autecology and behaviour of these cryptic species may also contribute to their co-occurrence: Pm III has the highest instantaneous fecundity [42], and also disperses at considerably lower population densities than other *Litoditis* species, in particular Pm I [41]. These characteristics suggest a life strategy with extreme colonization-extinction dynamics in Pm III. Nevertheless, the fact that we never found Pm III in high relative abundances may indicate that we did not sample its preferred habitat(s), which could for instance be decomposing *Fucus* spp. instead of the living *Fucus* spp. which were used in this study.

### Cryptic species composition varies among different algal structures

Not only did the cryptic species composition of *L*. *marina* differ among different species of macroalgae, it also varied among different structures of *F*. *spiralis* and *F*. *vesiculosus* for both stages of nematodes (Fig 3). On *F*. *spiralis*, Pm I was present on all algal structures, but was proportionally the most abundant on the thalli. In contrast, Pm II was only present on the receptacula and Pm III was found in higher proportions on the non-fertile tips. On *F*. *vesiculosus*, Pm I largely dominated the receptacula and bladders, while Pm II had high proportions on the thalli, and Pm III had a higher relative abundance on the receptacula compared to other structures of *F*. *vesiculosus*. Hence, a single macroalga clearly forms a spatially heterogenous habitat, which may be related to the presence of different microbial populations on different algal structures, as demonstrated for *Laminaria saccharina* [59].

Apart from the possible influence of microbial communities, *F*. *spiralis* and *F*. *vesiculosus* also have different physical features which may affect the cryptic species composition, either directly or through effects on the microbial biofilms. Functionally relevant characteristics include texture, colour, toxicity, and smell and taste [60]. Macroalgae may have physical properties which facilitate attachment of some species of bacteria while suppressing that of other strains [60]. Receptacula, the swollen reproductive tips of *Fucus*, appeared to be an ideal habitat for nematodes, probably because of the secretion of slimy mucus which may provide a rich food source for bacteria [64] and hence a high food availability for *L*. *marina*. However, we did not find the same nematode species composition on the receptacula of both *Fucus* species. All three cryptic species occurred on the receptacula of *F*. *spiralis*, albeit that Pm III was only found at low frequency. On *F*. *vesiculosus* receptacula, Pm I and low proportions of Pm III were still present, but no Pm II. Instead, Pm II largely dominated the thalli of *F*. *vesiculosus* where it also reached its highest relative abundance. These results need to be cautiously interpreted, since we only sampled the surfaces of the receptacula, while recent work demonstrates that *L*. *marina* also has the ability to hide inside receptacula and bladders [65].

Non-fertile tips, in contrast, later develop into receptacula, but they do not yet secrete slimy substances. Furthermore, the tips of *Fucus* can exhibit an anti-fouling strategy by periodically shedding surface cell layers [66], which could interfere with the ability of organisms to become ‘permanently’ established on this structure. We found high relative abundances of Pm I, but no Pm II on the tips of *Fucus*. Interestingly, the proportion of Pm III was significantly higher on non-fertile tips than on other structures of *F*. *spiralis*. The presence of this species on these ‘unstable’ tips agrees with its high colonization ability—the fastest disperser [41] with higher instantaneous fecundity compared with the other cryptic species [42]. It is also noteworthy that Pm III was consistently found in higher proportions on the structures from which Pm II was absent, i.e. on the non-fertile tips of *F*. *spiralis* and on the receptacula of *F*. *vesiculosus*; vice versa, Pm II was most abundant on the thalli of *F*. *vesiculosus* and the receptacula of *F*. *spiralis*, structures from which Pm III was absent. In laboratory experiments, Pm I and Pm III were found to be competitively superior over Pm II [43], but competition among cryptic species may also change when environmental conditions and/or the presence of other competitors change [42,44,67].

### Cryptic species composition varies between sampling months

The cryptic species composition of *L*. *marina* also varied between November and April (Figs 2 and 3). Seasonal fluctuations of *L*. *marina* in the Paulina area are in line with an earlier study which showed that Pm I was the most abundant species in every season, while Pm II was mostly found in autumn and Pm III was most frequent during summer but scanty in spring [39]. Correspondingly, we found Pm I at high relative abundances in both autumn (November) and spring (April), while Pm II was prominently present in autumn but nearly absent in spring. Pm III did not exhibit differences between months, probably because it occurred in consistently low relative abundances in our study. In laboratory experiments, Pm I did not exhibit clear temperature preferences in the range of 15 to 25°C, while Pm II preferred lower temperatures and Pm III performed better at higher temperatures [42]. These results correspond well with the results of our field sampling.

But were the different proportional abundances of the cryptic species of *L*. *marina* at different habitats (i.e. different algal species and different algal structures) consistent over time? This was only partly true. Pm II was consistently found in higher proportions on *Ulva* sp. than on *Fucus* species. We also observed a clearly higher proportional abundance of Pm I on *Fucus* species compared to *Ulva* in both months. For adults, temporal differences in the cryptic species composition were not consistent across algal species and vice versa. Lower relative abundances of Pm II on *Ulva* in April than in November may be related to the seasonality of this alga since *Ulva* was considerably less prominently present in April. Furthermore, the cryptic species of *L*. *marina* adults and juveniles showed differential proportional abundances on different algal structures on the same algal species in November, but we did not find such significant differences in April (Fig 3). This indicates that the species composition of *L*. *marina* is not only shaped by microhabitat ‘preferences’ but also by environmental conditions, and that there is a complex interaction between biotic components (macroalgae–microbial biofilms—nematodes) and abiotic factors. Abiotic factors, such as temperature and salinity [42,43], may not only impact the cryptic species but also the microbial communities on macroalgae, as strong seasonal shifts have been observed on the microbiota of *F*. *vesiculosus* and *Ulva intestinalis* [61].

### Microhabitat differentiation can partially explain the sympatric distribution of the *L*. *marina* complex

Our study shows that microhabitat partitioning can partially explain the co-occurrence of *L*. *marina* in the field. They can co-occur within the Paulina area by occupying different algal species, and even co-occur within a single macroalga by occupying different algal structures. Sympatric distribution of cryptic species driven by microhabitat differentiation has previously been highlighted for other invertebrate organisms, including Gastropoda [30, 55] and Amphipoda [15,16,68]. Microhabitat partitioning of sympatrically distributed cryptic species may probably be driven by interspecific competition for food or space causing ecological displacement [30], differential food preferences of the cryptic species [69], distinct environmental conditions in each microhabitat [30,70], predator pressure [71], or through competitive ability-predation risk trade-off [16]. For the *L*. *marina* complex, different algal species and algal structures may provide different food resources and/or reflect different ranges of abiotic conditions leading to microhabitat partitioning. This mechanism may weaken the competition between the cryptic species. Since temporal dynamics was found to play a role in this microhabitat segregation, both factors may offer several niches allowing co-occurrence of the cryptic species in the field. In ephemeral habitats such as the macroalgae inhabited by *L*. *marina*, dispersal may also be extremely important to be able to transfer to a favorable site [65]. Active dispersal does occur in this cryptic species complex [41] and may be an important factor which influences their microhabitat differences. If species disperse to a more ‘preferred’ microhabitat, then they can co-occur within a single area or even within a single macroalga.

Nevertheless, the cryptic species of *L*. *marina* were also encountered together at a very small spatial scale in some cases wherein Pm I, Pm II and Pm III occupied one particular algal structure, for instance in the case of the receptacula of *F*. *spiralis*. This might denote transitional stages before the outcompetition of the other species [70] or before they disperse to a more ‘preferred’ microhabitat. Alternatively, this may also indicate ecological similarity/equivalence of the cryptic species suggesting potential significance of both niche differences and equalizing effects (neutrality) in the co-occurrence of *L*. *marina*. Species that co-occur should be more similar species to one another than they are to species present under other ecological features because they respond similarly to environmental conditions [9]. The competitive intransitivity observed in *L*. *marina*, in such a way that cryptic species cannot be ordered hierarchically, may also represent ecological equivalence as a mechanism for coexistence [65]. It has been underlined that the presence of niche partitioning does not negate the importance of ecological equivalence, and that all ecological systems are probably somewhere between these endpoints- neither completely niche-structured nor completely neutral [72]. Therefore, a combination of different mechanisms to achieve coexistence of cryptic species cannot be excluded.

## Conclusions

Using real-time qPCR, we demonstrate that the cryptic species composition of the *Litoditis marina* complex on macroalgae varies among different species of macroalgae, but also among different structures of a particular algal species, which indicates that a single macroalga represents a spatially heterogeneous habitat. Different algal species and algal structures may provide different food resources and/or reflect different ranges of environmental conditions, leading to microhabitat partitioning in the *L*. *marina* complex. Nevertheless, the cryptic species composition of *L*. *marina* on different microhabitats was also subject to temporal shifts. This suggests that both microhabitat differences and temporal dynamics may offer several niches for *L*. *marina*, hence providing ample opportunities for co-occurrence of the cryptic species in the field.

## Supporting information

S1 Table Pair-wise test results for PERMANOVA analysis on the significant effect of sampling time (nested in month) on *Fucus spiralis* for juveniles and on *Fucus vesiculosus* for both stages. Significant differences (*P*<0.05) are highlighted in bold.
(DOCX)
