# Supplementary material for: Living apart-together: Microhabitat differentiation of cryptic nematode species in a saltmarsh habitat
Source: PLoS One. 2018 Sep 27;13(9):e0204750. doi: 10.1371/journal.pone.0204750 (PMC6160205; doi:10.1371/journal.pone.0204750)
Supplement: S1 Table — Significant differences (P<0.05) are highlighted in bold. (DOCX) [file pone.0204750.s001.docx]

Living apart-together: Microhabitat differentiation of cryptic nematode species in a saltmarsh habitat

Rodgee Mae Guden^1,2,*^, Anna-Maria Vafeiadou^1,3^, Nele De Meester^1^, Sofie Derycke^1,4^, Tom Moens^1^

*^1^Ghent University, Marine Biology Lab, Ghent, Belgium*

*^2^Mindanao State University- Iligan Institute of Technology, Iligan, Philippines*

*^3^Aristotle University of Thessaloniki, Biology Department, Thessaloniki, Greece*

*^4^Institute for Agricultural and Fisheries Research, Aquatic Environment and Quality, Oostende, Belgium*

*Corresponding author:

T: +32-(0)9-264 85 23

[rodgeemae.guden@ugent.be](mailto:rodgeemae.guden@ugent.be)

**S1 Table.** **Pair-wise test results for PERMANOVA analysis on the significant effect of sampling time (nested in month) on *Fucus spiralis* for juveniles and on *Fucus vesiculosus* for both stages. Significant differences (*P*<0.05) are highlighted in bold.**

| ***Fucus spiralis*** | | **Juveniles** | | | |  |  | |  | |  | | |
| --- | --- | --- | --- | --- | --- | --- | --- | --- | --- | --- | --- | --- | --- |
|  | F | | | *P*  value | *P*  adjusted |  |  | |  | | |  |  |
| 1:November vs 2:November | 2.81 | | | 0.088 | 0.53 |  |  | |  | | |  |  |
| 1:November vs 1:April | 21.09 | | | 0.0005 | **0.003** |  |  | |  | | |  |  |
| 1:November vs 2:April | 11.47 | | | 0.0015 | **0.009** |  |  | |  | | |  |  |
| 2:November vs 1:April | 5.45 | | | 0.02 | 0.12 |  |  | |  | | |  |  |
| 2:November vs 2:April | 1.74 | | | 0.19 | 1.00 |  |  | |  | | |  |  |
| 1:April vs 2:April | 1.56 | | | 0.21 | 1.00 |  |  | |  | | |  |  |
|  |  | | |  |  |  |  | |  | | |  |  |
| ***Fucus vesiculosus*** | | **Adults** | | | |  | **Juveniles** | | | | | | |
|  | F | | *P*  value | | *P*  adjusted |  | F | *P*  value | | *P*  adjusted | | | |
| 1:November vs 2:November | 0.86 | | 0.36 | | 1.00 |  | 0.78 | 0.47 | | 1.00 | | | |
| 1:November vs 1:April | 4.54 | | 0.038 | | 0.23 |  | 8.09 | 0.013 | | 0.075 | | | |
| 1:November vs 2:April | 3.61 | | 0.054 | | 0.32 |  | 3.90 | 0.052 | | 0.31 | | | |
| 2:November vs 1:April | 1.91 | | 0.19 | | 1.00 |  | 8.16 | 0.0046 | | **0.028** | | | |
| 2:November vs 2:April | 1.29 | | 0.24 | | 1.00 |  | 3.74 | 0.039 | | 0.23 | | | |
| 1:April vs 2:April | 0.09 | | 0.83 | | 1.00 |  | 2.56 | 0.13 | | 0.77 | | | |
